# Editorial: Emerging and reemerging neglected tropical diseases: epidemiology, transmission, mitigation, and vaccines and chemotherapy advancements

**DOI:** 10.3389/fphar.2025.1545801

**Published:** 2025-03-31

**Authors:** Ranjan K. Mohapatra, Snehasish Mishra, Venkataramana Kandi, Chandra Sekhar Sirka, Lawrence Sena Tuglo

**Affiliations:** ^1^ Department of Chemistry, Government College of Engineering, Keonjhar, Odisha, India; ^2^ School of Biotechnology, KIIT Deemed-to-be University, Bhubaneswar, Odisha, India; ^3^ Department of Microbiology, Prathima Institute of Medical Sciences, Karimnagar, Telangana, India; ^4^ Department of Dermatology, All India Institute of Medical Sciences, Bhubaneswar, Odisha, India; ^5^ Department of Nutrition and Dietetics, School of Allied Health Sciences, University of Health and Allied Sciences, Hohoe, Ghana

**Keywords:** neglected tropical diseases, epidemiology, transmission, mitigation strategy, vaccines and chemotherapy

## Introduction

**FIGURE 1:**
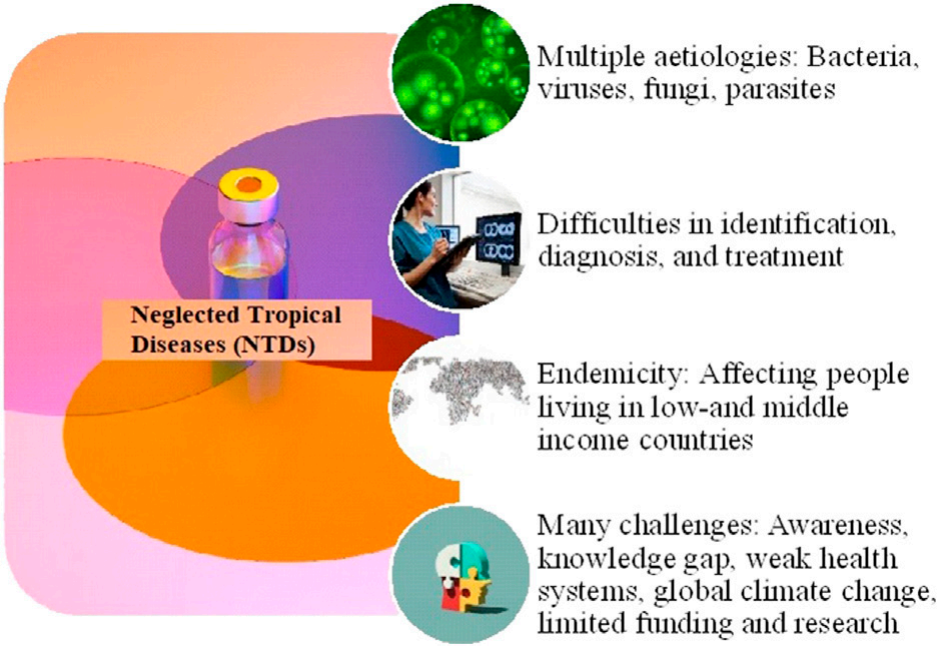
The numerous perennial social, economic and technical challenges facing NTD control.

Neglected tropical diseases (NTDs) are infectious diseases caused by bacteria, viruses, fungi and parasites, including ectoparasites like mites and fleas (Mohapatra et al., 2024; Kutikuppala et al., 2023; WHO, 2024). NTDs are an ongoing challenge to global public healthcare and community health. The main reason why such diseases remain collectively neglected is that they are considered “diseases of the poor”, primarily people in low- and middle-income countries (LMICs) with modest purchasing capacity. As a result of this, the commercial diagnostic, therapeutic and prophylactic efforts by the pharmaceutical companies are only skeletal as they do not envision a profitable market. Thus, although treatable, these diseases ultimately manifest as terminal diseases of the have-nots. The World Health Organisation (WHO) has compiled a list of the world’s most prevalent NTDs and updates it from time to time. Currently, there are 21 aetiologically, epidemiologically and clinically unique diseases (or groups of diseases) listed as NTDs by the WHO (Malecela and Ducker, 2021). The WHO has devised public health strategies and proposed a roadmap to eliminate NTDs by 2030 (WHO, 2021). However, as the majority of the NTD-affected subjects live in financially constrained third-world countries, this seems to be an uphill task and achieving it is by no means easy. Lack of NTD-related awareness and limited diagnostic resources in these regions severely affect their foolproof identification. This is evidenced by the constantly increasing number of diseases that fit into the WHO criteria for NTDs. Various NTDs re-emerged in the wake of the coronavirus disease 2019 (COVID-19) pandemic by the severe acute respiratory syndrome coronavirus-2 (SARS-CoV-2), which hit the healthcare infrastructure hard worldwide and exposed its underbelly. This suggests the need for increased collaborative research efforts to improve the diagnosis, control and prevention of NTDs possibly through special benevolent funding by the global health agencies and charitable trusts for the sake of humanity. In the wake of the above, there is a constant need to keep the scientific community informed and updated about these “diseases of the poor”, and to sensitise the numerous global stakeholders to the universal wellbeing and economic consequences of NTDs far beyond the international borders. This editorial highlights the contributions of the various authored articles that are included in our Research Topic dedicated to research topics on NTDs. Figure 1 summarises the numerous perennial social, economic and technical challenges facing NTD control for readers’ better understanding. This Research Topic was an attempt to decipher the best-case scenario for devising a unified global response to contain diseases across borders and save precious human lives.

## Discussion

The global impact of a tick-borne apicomplexan bovine babesiosis parasite, *Babesia bovis* has been underestimated, particularly in tropical and subtropical regions. Using the Texas T2Bo strain of *Babesia bovis* in a US study, Cardiollo et al. compared the efficacy of imidocarb dipropionate (ID) and buparvaquone (BPQ) *in vitro*. The study evaluated the effect of the two drugs at varying concentrations against laboratory-cultured infected cells. The authors revealed that the parasite could be completely eliminated at 150 nM of BPQ and 300 nM of ID, and thus that BPQ was superior in efficacy to ID. Thus the data suggest that BPQ is effective and appears to be superior to ID as a first-line drug to treat the global threat of bovine babesiosis.

The 2015 Zika virus (ZIKV) outbreak in Brazil attracted the global research community as the epidemic spread throughout the Americas. Over 5,00,000 ZIKV-confirmed cases had been reported in the Americas by the end of 2016. Due to its serious threat to public health, the WHO declared ZIKV infection a public health emergency of international concern (PHEIC). To date, 86 countries and territories have reported ZIKV infection, while there are no effective treatments or vaccines for it. Among several vector-borne viral infections, ZIKV is a mosquito-borne infection transmitted through the bite of *Aedes aegypti*. ZIKV belongs to the Flaviviridae family of RNA viruses, which also includes other human infectious viruses such as yellow fever virus, dengue virus and Japanese B encephalitis. Feng reviewed a list of drugs and natural compounds with alleged antiviral activity against ZIKV. Among these, protein inhibitor groups such as LAS 52154459, LAS 52154463 and LAS 52154474 were found to be the potential candidates as they could efficiently contain the disease and were less toxic. The review summarised the role of NS2B-NS3 protease inhibitors such as peptide derivatives, repurposed drugs and synthetic small molecules that limit viral development and replication. NS5 protein inhibitors such as MTase inhibitors, RdRp inhibitors, and nucleoside analogues also were reviewed for their anti-ZIKV potential. As a result, continued research in this area to develop ZIKV medications is recommended as the most promising and effective approach.

Dermatomycosis or dermatophytosis is a superficial fungal infection condition of the skin, hair and nails. Despite the mildness of the clinical presentations, such infections are severely debilitating. Adequate knowledge of the predisposing factors behind such infections and the preventive measures could contribute to low infection rates. In this context, Dhakal et al. evaluated the knowledge, hygiene practices and prevalence of dermatophytosis among prisoners in Nepal in their original survey-based study. In this cross-sectional study, 184 Nepali prisoners were included whose dermatophytosis data were collected using a validated questionnaire. The results demonstrated that the knowledge of dermatophytosis among prisoners was moderate. The results also revealed a significant knowledge gap regarding the transmission of the infection and the recommended preventive measures. Despite awareness of available treatments, there were misconceptions about reinfection and the role of personal hygiene in controlling the spread of infection. Given the crowded and sometimes overcrowded nature of prisons, the authors concluded that it is important to implement measures to improve the understanding and awareness of dermatophytosis and preventive strategies among this population.

*Opisthorchis viverrine* is a helminthic parasite fluke of the liver belonging to the Platyhelminthes group. It is one of the many NTDs that are endemic to the Greater Mekong Subregion of Thailand. Humans infected with *O*. *viverrine* would suffer from go on to develop cholangiocarcinoma. Although the infection could be treated with the anti-helminthic praziquantel, reinfection with *O*. *viverrine* due to the development of antimicrobial resistance severely limits the treatment option. Therefore, focused research directed toward the development of vaccines to prevent this parasitic infection is essential. Kafle and Ojha elucidated the application of Omics approaches and computational tools in the development of advanced countermeasures against *O*. *viverrine* infection. The applications of cutting-edge tools and techniques based on Omics approaches such as genomics (genome repeat and non-coding element identification, sequencing, genome assembly, protein-coding gene identification and functional annotation), transcriptomics (differential gene expression analysis, RNA sequencing, transcriptome assembly and functional annotation), and proteomics (protein extraction, mass spectroscopy, protein identification, immunoreactive band excision and functional annotation), and the potential therapeutic targets were discussed in detail in their report. The approaches based on computational tools such as Artificial Intelligence will be very helpful to accelerate NTD-related research and discovery of novel biologics with limited available funds, opening new vistas to effectively combat not only such neglected diseases as infection with the carcinogenic human liver fluke *O*. *viverrine* but also other NTDs of global concern.

Arboviral infection is a significant threat to community health due to its associated morbidity and mortality. Arboviral infectious diseases are transmitted through the active involvement of arthropod vectors such as mosquitoes. These infections usually spread during the monsoon season, when the mosquito vectors breed and multiply exponentially. Compounded by climate change and increasing global movements, Italy faces a growing threat of arboviral infections due to the presence of competent mosquito vectors such as *A. albopictus*. Branda et al. have discussed the ArboItaly (Arbovirus in Italy) and the utility of computerised tools in this context, to effectively carry out genomic surveillance of arboviruses in Italy. Their suggested approaches include data Research Topic, classification, dataset processing and online publication of the results of the analyses for general public access and use by the scientific community. The authors discussed in detail the environmental and socioeconomic factors responsible for the spread of arboviral infections, the control of these infections through integrated strategies and the promotion of research advances in the development of vaccines against them. They observe that the chikungunya virus (CHIKV) vaccine currently in Phase I clinical trials prepared using the pre-membrane (prM) and envelope (E) glycoproteins of CHIKV is more than 90% effective, and a recombinant yellow fever vaccine using the same proteins shows similar efficacy. Although Dengvaxia^®^ (CYD-TDV) developed by Sanofi Pasteur and Qdenga^®^ (TAK-003) developed by Takeda are vaccines against the Dengue virus (DENV) and are licensed for human use, there is no approved vaccine against CHIKV for human use. Therefore, enhanced vector control measures and public healthcare interventions to curb the widespread transmission of arboviral infections particularly in high-risk regions are urgently needed, especially in the absence of effective preventive and therapeutic countermeasures.

Among several other arboviral infections, DENV is extremely prevalent in tropical and subtropical Asia and the Americas. DENV is transmitted by the bite of the *Aedes aegypti* mosquito. There are four types of DENV (DENV 1–4) around the globe, of which DENV-2 serotype infection is fatal. With over 100 million illnesses and 25,000 deaths annually, it has significantly impacted global healthcare and wellbeing. Hossain et al. evaluated the phytochemical compounds sourced from *V. cinerea* for their antiviral properties against DENV. Based on the thermodynamic properties, electrostatic potentials and the molecular screening of *β*-amyrin, *β*-amyrin acetate, chrysoeriol, isoorientin and luteolin as test compounds, these could potentially inhibit the NS1 protein of DENV-2. This study demonstrated that there was no cytotoxicity, hepatotoxicity, immunotoxicity, mutagenicity or carcinogenicity of the five test compounds that they studied. The authors suggest through their article that chrysoeriol is the most effective agent against DENV-2 and that it could be evaluated further to manage DENV-2 infections.

Since its discovery in 2019, the SARSCoV-2 virus has continued to exist and circulate. Although it resulted in severe morbidity and mortality in the initial stages when information about the aetiological agent SARS-CoV-2 was scarce, the early introduction of vaccines could control its further spread and later reduce the severity of the disease. As evidenced by variants such as Alpha, Beta, Delta, Omicron, etc., SARS-CoV-2 is constantly evolving and thriving with its genetic variants. COVID-19 patients have frequently reported post-recovery sequelae, termed “long COVID-19”, where they suffer from chronic symptomatic manifestations in internal organs such as the lungs. Chhotaray et al. evaluated the cardiovascular complications associated with SARS-CoV-2 infection by the JN.1 subvariant. JN.1 was identified in September 2023 and was initially labelled as a variant of interest (VoI) by the WHO. It was more prevalent in Canada, France, Sweden, Singapore, the United Kingdom and the United States. The unique mutations observed in JN.1 are R3821K in ORF1a, L455S in the spike protein, and F19l in ORF7b. Evidence suggests that since SARS-CoV-2 infection is facilitated by the ACE2 receptors in human cells, it affects the renin-angiotensin system (RAS) which is also involved in hypertension, and it could result in complications of the cardiovascular system. The authors also suggest that the emergence of novel variations and their severe outcomes should be closely monitored. They also emphasise the development of novel monoclonal antibodies and next-generation vaccines to counter the virus’ adverse effects.

With the emergence of mpox as an endemic disease in the Democratic Republic of the Congo and developing as the next potential pandemic, the focus of public health administrators, researchers and clinicians has shifted to MPXV (the causative agent of mpox infection). MPXV, which causes infections in vertebrate hosts such as humans, cattle and monkeys, belongs to the poxvirus lineage consisting of Orthopoxviruses. Of the MPXV clades, Clade I is found to be more pathogenic than Clade II. However, Clade I was restricted to West African countries until recently when the virus was first reported. Clade II reemerged in 2022 spreading to the United States, and later spreading to more than 120 countries worldwide The clear evidence of the recent spread of Clade I beyond the endemic regions and the emergence of the novel Clade Ib lineage is a global public health threat, and certainly a wake-up call. In this context, Srivastava et al. reviewed the geographic distribution, transmission characteristics, mutation rate and public healthcare implications of all the MPXV clades. They explored the benefits of artificial intelligence in the diagnosis and surveillance of MPXV infections which could assist in predicting and preventing outbreaks in the future.

The Research Topic of articles in this Research Topic on NTDs certainly addresses the knowledge gaps and enhances the comprehension to effectively mitigate the transmission of these NTDs, particularly in low- and middle-income countries (LMICs). Furthermore, the use of advanced computational tools such as DL, ML and AI, along with “One Health” approaches, could facilitate the forging of solutions to contain these diseases. The many other infectious NTDs (such as tuberculosis) and non-infectious NTDs (such as noma) that are frequently encountered could arguably find more promising solutions through such investigative research efforts. The Editors envision and hope to produce a new Research Topic covering some of these diseases at a later date.
